# Supplementary material for: Splice-Junction-Based Mapping of Alternative Isoforms in the Human Proteome
Source: Cell Rep. Author manuscript; Available in PMC 2020 Jan 15. (PMC6961840; doi:10.1016/j.celrep.2019.11.026)
Supplement: 2 [file NIHMS1546469-supplement-2.zip › DF1/PXD006675/LeftAtrium/LeftAtrium_25_NDUFA5_DSGLVGLAVCNTPHER.pdf]

A

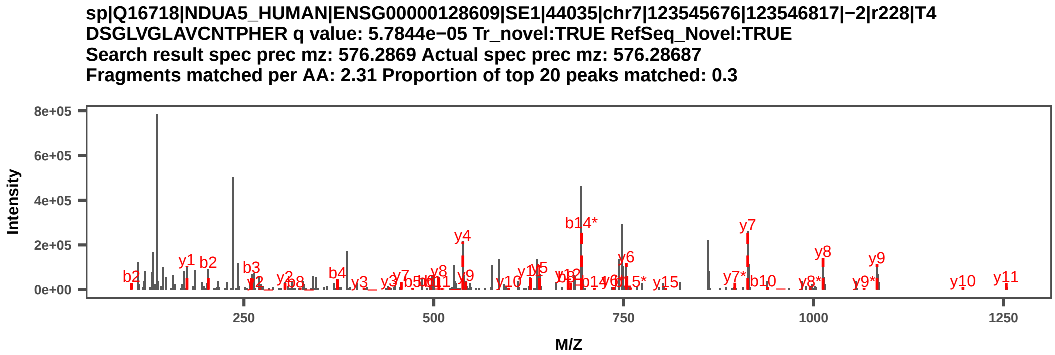
sp|Q16718|NDUA5_HUMAN|ENSG00000128609|SE1|44035|chr7|123545676|123546817|−2|r228|T4
DSGLVGLAVCNTPHER q value: 5.7844e−05 Tr_novel:TRUE RefSeq_Novel:TRUE
Search result spec prec mz: 576.2869 Actual spec prec mz: 576.28687
Fragments matched per AA: 2.31 Proportion of top 20 peaks matched: 0.3
Intensity
M/Z
b14*
y7
y4
y8
y9
y6
y1
b2
b3
b4
y10
y11
y15
y7*
y8*
y9*
b10
0e+00
2e+05
4e+05
6e+05
8e+05
250
500
750
1000
1250

B

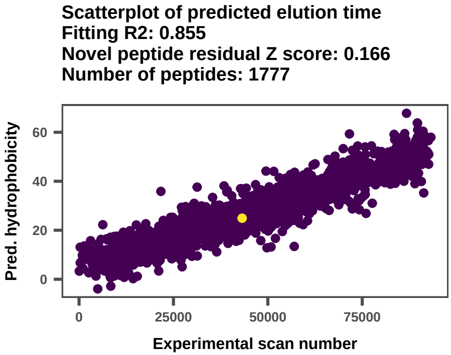
Scatterplot of predicted elution time
Fitting R2: 0.855
Novel peptide residual Z score: 0.166
Number of peptides: 1777
Pred. hydrophobicity
Experimental scan number
0
20
40
60
0
25000
50000
75000

C

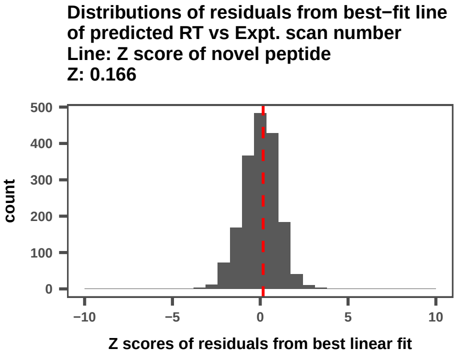
Distributions of residuals from best−fit line
of predicted RT vs Expt. scan number
Line: Z score of novel peptide
Z: 0.166
count
Z scores of residuals from best linear fit
0
100
200
300
400
500
−10
−5
0
5
10
